# The Impact of Official Development Aid on Maternal and Reproductive Health Outcomes: A Systematic Review

**DOI:** 10.1371/journal.pone.0056271

**Published:** 2013-02-22

**Authors:** Emma Michelle Taylor, Rachel Hayman, Fay Crawford, Patricia Jeffery, James Smith

**Affiliations:** 1 Centre of African Studies, University of Edinburgh, Edinburgh, United Kingdom; 2 International NGO Training and Research Centre, Oxford, United Kingdom; 3 Centre for Population Health Services, University of Edinburgh, Edinburgh, United Kingdom; 4 Sociology, University of Edinburgh, Edinburgh, United Kingdom; The University of Adelaide, Australia

## Abstract

**Background:**

Progress toward meeting Millennium Development Goal 5, which aims to improve maternal and reproductive health outcomes, is behind schedule. This is despite ever increasing volumes of official development aid targeting the goal, calling into question the distribution and efficacy of aid. The 2005 *Paris Declaration on Aid Effectiveness* represented a global commitment to reform aid practices in order to improve development outcomes, encouraging a shift toward collaborative aid arrangements which support the national plans of aid recipient countries (and discouraging unaligned donor projects).

**Methods and Findings:**

We conducted a systematic review to summarise the evidence of the impact on MDG 5 outcomes of official development aid delivered in line with Paris aid effectiveness principles and to compare this with the impact of aid in general on MDG 5 outcomes. Searches of electronic databases identified 30 studies reporting aid-funded interventions designed to improve maternal and reproductive health outcomes. Aid interventions appear to be associated with small improvements in the MDG indicators, although it is not clear whether changes are happening because of the manner in which aid is delivered. The data do not allow for a meaningful comparison between Paris style and general aid. The review identified discernible gaps in the evidence base on aid interventions targeting MDG 5, notably on indicators MDG 5.4 (adolescent birth rate) and 5.6 (unmet need for family planning).

**Discussion:**

This review presents the first systematic review of the impact of official development aid delivered according to the Paris principles and aid delivered outside this framework on MDG 5 outcomes. Its findings point to major gaps in the evidence base and should be used to inform new approaches and methodologies aimed at measuring the impact of official development aid.

## Introduction

In 2000, United Nations member states signed up to the Millennium Development Goals (MDGs), a set of eight international development targets intended to catalyse development and reduce global poverty. To date progress towards these goals has been uneven. Of particular concern is Millennium Development Goal 5 (MDG 5), which aims to improve maternal and reproductive health by reducing the maternal mortality ratio (MMR) by 75% and creating universal access to reproductive healthcare by 2015. Current estimates suggest that this initiative is behind schedule. Only 23 countries out of a surveyed 181 are likely to meet the MMR target on time despite increasing volumes of official development aid being provided by donors [1], [2]. There is concern, therefore, that not all the aid targeting MDG 5 is reaching the countries in the greatest need or being delivered in an effective manner [2], [3].

The adoption of the MDGs came at the end of a decade in which the purpose and usefulness of official development aid had come under increased scrutiny. The changing geopolitical climate of the 1990s, coupled with the poor results of decades of work and billions of dollars aimed at improving social and economic conditions in poor countries, led to a questioning of the usefulness and effectiveness of overseas development aid. In the 2000s, a series of global high-level fora, involving international institutions, governments of developed and developing countries, and aid agencies, was held to debate the provision of aid and its management. These resulted in global commitments aimed at improving the effectiveness of aid. Central to these was the *Paris Declaration on Aid Effectiveness* which was signed in 2005 by over 100 agencies and governments [4].

**Figure 1:**
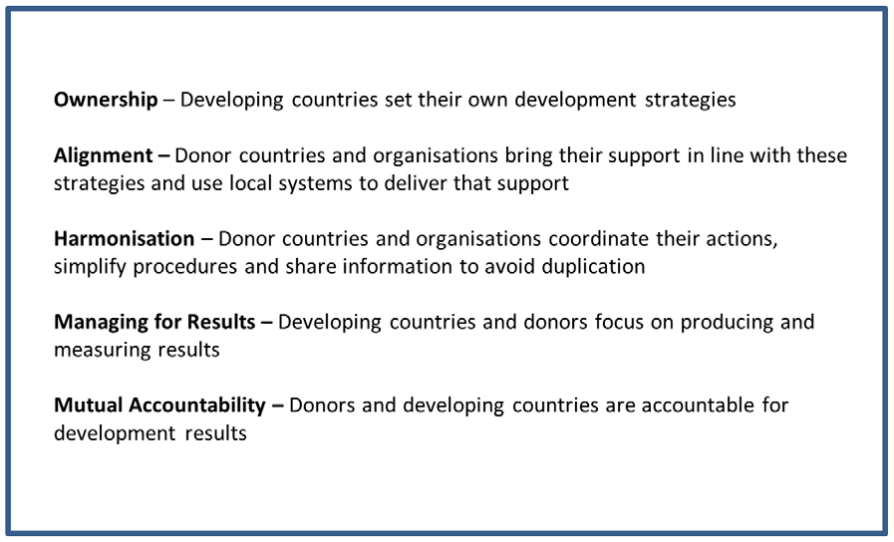
Paris Principles.

**Figure 2:**
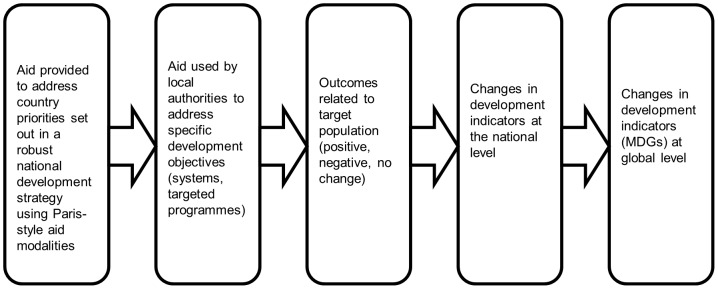
Flow diagram depicting the theorised impact of aid delivered under the Paris principles.

**Figure 3:**
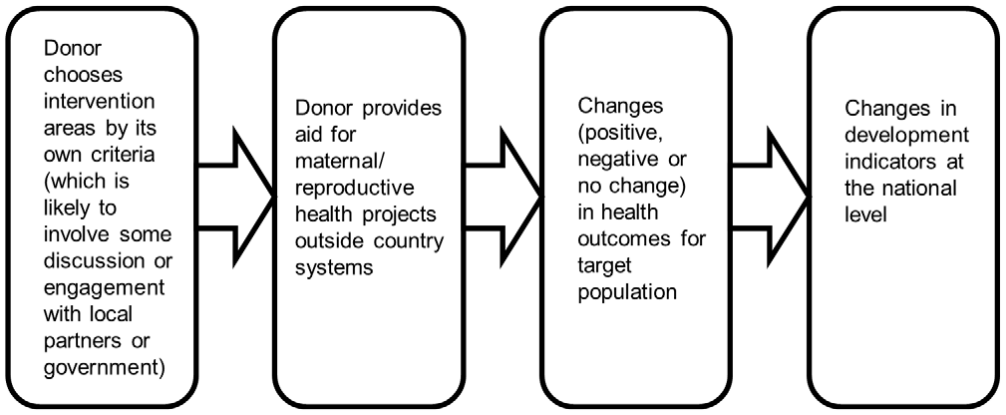
Flow diagram depicting the theorised impact of aid not delivered under the Paris principles.

The adoption of the *Paris Declaration* in 2005 represented the commitment of the international community to improve aid management and delivery. The Paris Declaration was a political statement, which set out guiding principles that signatories were expected to adopt in their delivery of and use of aid. The underlying theory was that aid delivered according to five principles (the Paris Principles) - ownership, alignment, harmonisation, managing for results and mutual accountability – would contribute to improved development outcomes by virtue of all partners working together to achieve the objectives set out in national development strategies (figure 1). This approach aimed to address the problems arising from donors funding multiple, unaligned projects outside the control, and sometimes even the knowledge, of the authorities of the country (figures 2 and 3). Various methods, indicators and tools were subsequently devised to track progress in the implementation of the Paris Principles, including country-level (donor and beneficiary) surveys and evaluation frameworks; and large-scale multi-country evaluations [5], [6].

The question of whether the revised aid agenda epitomised by the Paris Declaration – and its sister document the *Accra Agenda for Action* which was adopted in 2008 and reconfirmed the Paris Principles – is having an impact on MDG 5 is pertinent as the 2015 deadline approaches [7]. To date the majority of studies tracking the effect of the Paris Declaration have focused on shifts in the practice and management of aid – i.e. the processes around aid delivery - rather than on evaluating outcomes.

This systematic review was commissioned by the UK’s Department for International Development (DFID) in 2010, ahead of the 4^th^ High Level Forum on Aid Effectiveness which was held in Busan in 2011. It was one of a number of reviews commissioned as a pilot exercise to enhance the evidence base on the impact of development interventions and inform international development policy. This particular systematic review emerged from a desire to take the research base one step further than those studies exploring the implementation of the Paris Declaration, by focusing on studies which present robust evidence of the impact of interventions delivered in the context of the Paris Principles in bringing about changes in maternal and reproductive health outcomes. At the request of the commissioning body, the review also took a comparative approach - assessing the impact of aid not delivered according to the Paris Principles on maternal and reproductive health.

The objectives of the review are 1) to summarise the evidence of the impact on MDG 5 outcomes of delivering official development aid in line with Paris and Accra aid effectiveness principles and 2) to compare this with the impact of aid in general on MDG 5 outcomes. The review question was set by DFID.

## Methods

The protocol and full report for this systematic review are available online [8], [9].

### Eligibility Criteria

#### Participants

Studies had to refer to developing countries or developing regions of the world (Participants). In our review developing countries are those categorised as ‘Medium Human Development’ and ‘Low Human Development’ in the Human Development Index of 2009 [10].

#### Intervention

Studies had to report on official development aid delivered according to the Paris aid effectiveness principles (which we call ‘Paris style aid’); this could mean any aid intervention underpinned by some or all of the five Paris principles. However, as the Paris principles are relatively new (adopted in 2005), many studies on aid projects and programmes aimed at addressing maternal and reproductive health do not use this terminology or are not explicit about whether the aid in question would conform to these principles. Moreover, no set definitions exist to denote what does and does not constitute Paris style aid. In order not to lose relevant studies, we devised a categorisation system which places aid modalities in a hierarchy.

In our system, general budget support is the aid modality that most closely conforms to the Paris Principles as it is given directly to the central government of a recipient, with no directing of how it should be spent. It requires that the recipient has in place a robust national development strategy which is well managed and transparent. It is therefore underpinned by principles of ownership, alignment, mutual accountability and managing for results. Sector budget support and basket funds also adhere to the Paris Principles, but in a more constrained way, notably they are less ‘owned’ by the national government as donors retain considerable control over where aid is allocated, but are closely aligned with national plans, harmonised, and carrying strong respect for mutual accountability. Some types of project aid can also be considered to adhere to the Paris Principles, if they are sufficiently harmonised with that of other donors and if they are aligned with government plans, ideally with aid reported within the government budget (otherwise known as on-plan and on-budget). We might anticipate seeing this type of project aid within a sector-wide approach, where donors support a comprehensive sector policy led by the government. The aid provided by donors to a sector-wide approach can take any form.

**Figure 4:**
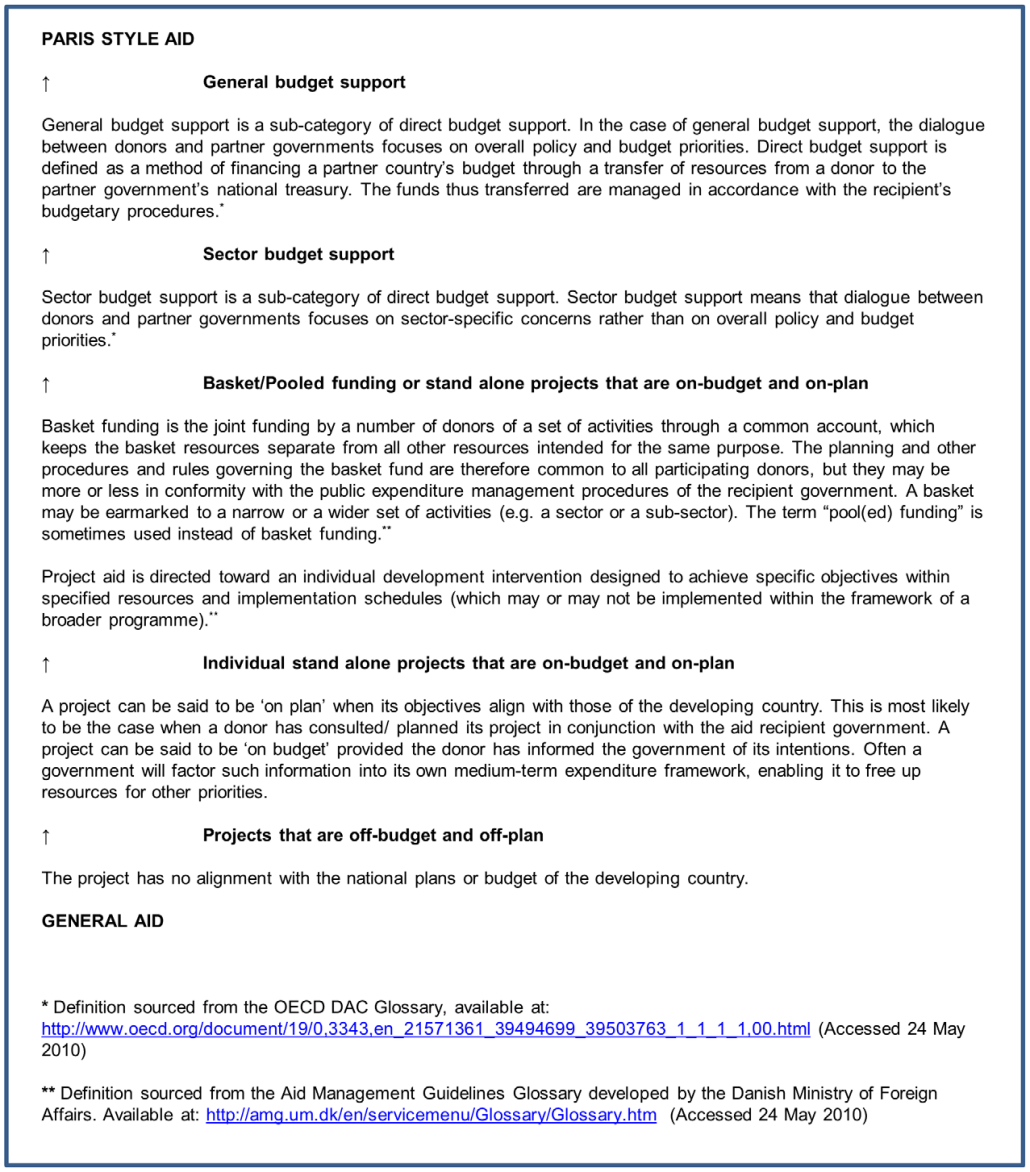
Hierarchy of aid modalities including definitions.

Other types of project aid cannot be said to adhere to the Paris Principles, namely when projects are managed and delivered outside country frameworks and financial systems (otherwise known as off-plan and off-budget). These we consider to be a proxy for non-Paris-style aid (i.e. ‘general aid’). See figure 4 for our aid hierarchy which includes detailed definitions of Paris style and general aid.

#### Comparison

Studies had to report on official development aid not delivered according to the Paris principles (‘general aid’), i.e. aid interventions that are managed and delivered outside country frameworks and financial systems. The difficulty here is that some interventions may appear or lay claim to being aligned or harmonised. The key element which distinguishes between Paris-style aid and general aid in our system is whether aid is ‘on-budget’ or not, i.e. reported within the budget of the recipient government.

#### Outcomes

Studies had to evaluate the effect of aid on at least one of the six MDG 5 target indicators (United Nations website for the MDG indicators. Available: http://mdgs.un.org/unsd/mdg/Host.aspx?Content=indicators/officiallist.htm. Accessed 2013 May 3):

1. MDG 5.1 maternal mortality rate or ratio;
2. MDG 5.2 proportion of births attended by skilled health personnel;
3. MDG 5.3 contraceptive prevalence rate among married women, aged 15–49;
4. MDG 5.4 adolescent birth rate;
5. MDG 5.5 ante-natal care coverage;
6. MDG 5.6 unmet need for family planning.

The success or otherwise of the aid intervention would be demonstrated by changes in the MDG 5 target indicators, backed up by evidence.

#### Study design

Studies had to present statistical evidence of the impact of aid on MDG 5 outcomes. Studies were categorised according to design as follows:

‘causal’ studies: these would present causal impact data and would be based on experimental (randomised) or quasi-experimental research design, which we defined as non-randomised designs used to test a causal hypothesis. ‘Causal’ studies would produce the strongest evidence of impact.
‘correlation’ studies: these were defined by us as observational studies which test an association between the intervention and the outcome recorded and would come from non-experimental designs. ‘Correlation’ studies would give weaker evidence possibly suggestive of impact.

### Search Strategy

We designed a search strategy that involved a round of systematic searching for potentially eligible studies.

We searched the following databases from 1990–2010 (this reflects the period during which a concerted effort was made to reform international aid management practices): Web of Science, Dissertations and Theses, Index to Theses, MEDLINE, EMBASE, Cinahl, Popline, Global Health Library (incorporating LILACS, AFRO, EMRO, PAHO, WHOLIS, WPRO), Econlit, IBSS, JOLIS, and IDEAS. Key organisation websites were trawled or, where feasible, searched using keyword searches (i.e. Google advanced searches): DFID, GFATM, OECD, PATH, USAID, UNIFEM, White Ribbon Alliance, World Bank, and World Health Organisation. Topic gateways were trawled or, where feasible, searched using keyword searches (i.e. Google advanced searches): ELDIS, BLDS, Aid Effectiveness Portal, and DFID Research 4 Development. Keyword searches were conducted using Internet search engines Google and Google Scholar. Reference lists were inspected from relevant existing evidence syntheses, systematic and literature reviews. Direct contact was made with authors and experts working in the fields of maternal health and aid which yielded specialist recommendations. Due to limited resources, no hand searching of journals was undertaken.

A full record of the search strategies used in this review is presented in the final report [9]. The search strategy used for Popline is presented here as an example:

**Popline** (www.popline.org searched 2010-08-08) (official development assistance/global health initiative*/global fund*/((aid/donor) & (disbursement*/commitment*/flow*/international/development/project*/program*))) & (maternal health/maternal health services/reproductive health/maternal mortality/family planning/contraceptive usage/adolescent pregnancy/(birth* & attend* & skill*)/(Millennium Development Goal* & 5)/MDG5/MDG 5).

### Study Selection

One reviewer (EMT) applied the eligibility criteria to the yield from the search activities beginning with the title and abstracts and, if the report was considered suitable, the full report.

### Data Extraction

Data were extracted by a single reviewer (EMT), using a coding tool designed specifically for the review (presented in the final report) [9]. Data were sought under the following headings: general information, study details, aid information, contextual information, MDG focus, data, study findings and additional comments, and study claims.

### Quality Assessment

A quality assessment was conducted by three reviewers (EMT, RH and FC). Disagreements were resolved by discussion.

**Figure 5:**
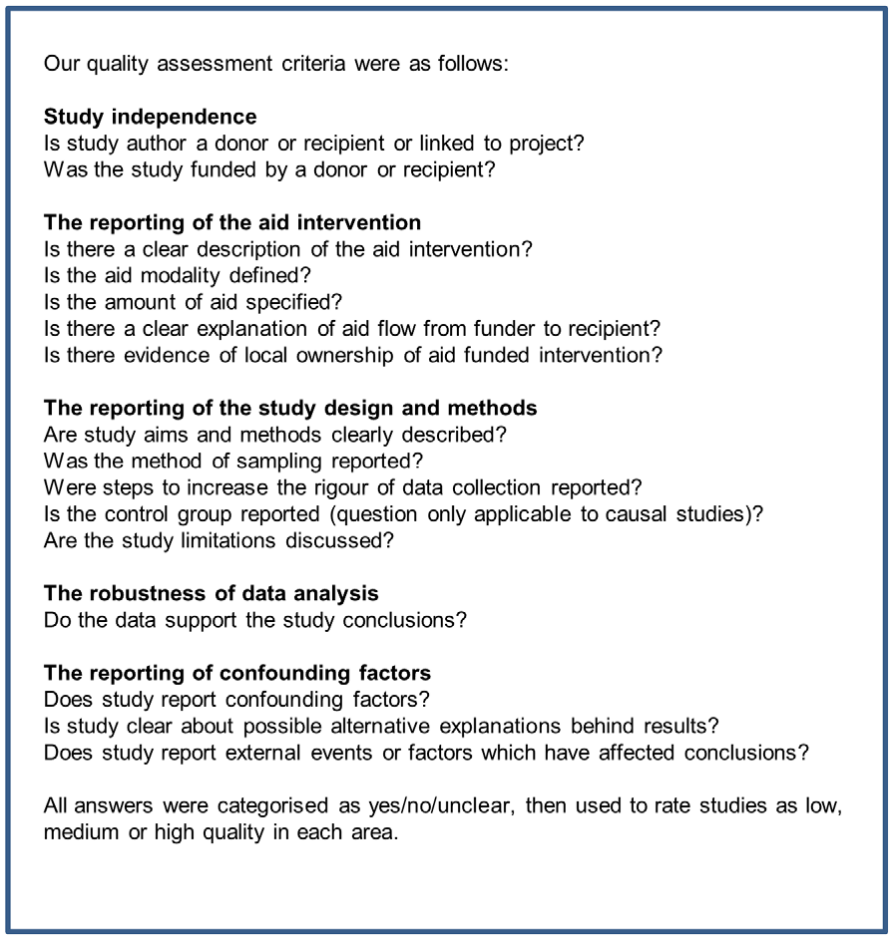
Quality assessment tool.

As no single approach to the assessment of quality and assessment of bias in studies evaluating the effect of international aid exists [11], we created a quality assessment tool, drawing on items used in previous reviews [12]–[15]. The tool posed questions to assess: study independence, the reporting of the aid intervention, the reporting of the study design and methods, the robustness of data analysis, and the reporting of confounding factors. All answers were categorised as yes/no/unclear, then used to rate studies as low, medium or high quality in each area (figure 5). The results were used to identify the risk of bias in each study (primarily with regards to study independence), potential weaknesses in the study design and findings, and for descriptive purposes, i.e. to determine the nature of the aid intervention described.

The findings of the quality assessment were used to divide studies into two pools. ‘Pool A’ contained studies on interventions which demonstrated adherence to some or all of the Paris principles, while ‘Pool B’ contained studies classified as general aid either because there was no indication of adherence to the Paris principles or because the information was too limited to make a sound judgement.

### Quantitative Data Synthesis

Where appropriate, we intended to re-calculate summary statistics for each study based on absolute numbers for each outcome extracted from the primary studies. Where a pooled estimate of data did not make practical sense, we planned to present absolute numbers for each study and calculate a measure of effectiveness with 95% confidence intervals. We intended to present data for each of the MDG 5 indicators. If these data were amenable, we would also pool estimates of effectiveness of aid for each of these variables. Tests for heterogeneity would be performed and where I2≥30% or a Q statistic of p = <0.1 was obtained a random effects model would be used. We also intended to perform sensitivity analyses based on the findings from the quality assessment process to compare the data derived from the highest quality studies with those studies found to be of poorer quality.

## Results

### Identified Studies

**Figure 6:**
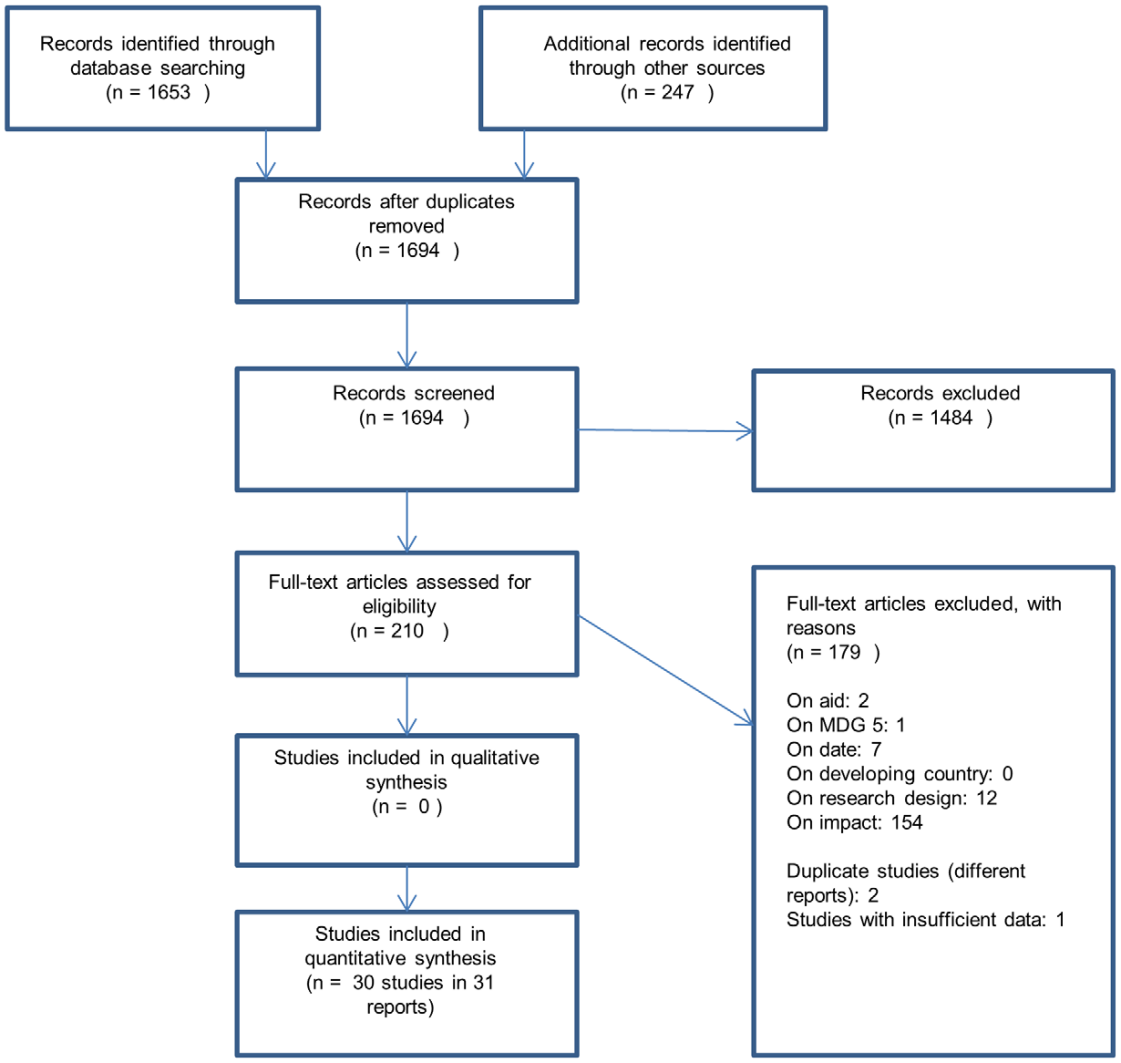
PRISMA flow diagram.

In total we identified 1900 citations, of which 211 were selected as potentially relevant to the review. 30 studies (in 31 reports) met all our inclusion criteria and were taken forward for synthesis (figure 6).

We found no existing systematic review on this topic, nor did we find any studies which answered the review question in its entirety, i.e. no studies compared the impact on the MDG 5 indicators of aid delivered using the principles set out in the Paris Declaration on Aid Effectiveness with aid delivered outside this framework.

Ten of the 30 studies concerned Paris-style aid (see table 1) [16]–[25]. They fall into the following aid sub-categories:

**Table 1:** Characteristics of Pool A Studies: Paris Style Aid.

Study	Study Design	Outcomes Measured	Programme/Project Name	Country	Aid Donor/s	Analysis of aid modality and management	Funding Amount	Year of Intervention
Barnett 2007 [16]	Correlation	MDG 5.2	Supporting three consecutive strategy papers (Country Strategy Paper - CSP – 2000–2005; Country Approach Paper - CAP – 2004–2008; Vision Paper 2004–2011)	Indonesia	UK’s Department for International Development	Intervention takes a harmonised approach with other donors (low intensity partnership with German agencies and UNICEF), so adherence to Paris Principles regarding harmonisation, and uses a mixture of aid modalities. There is limited information on other aspects of the Paris Principles, such as government ownership or use of national systems.	Average of USD 24.49 m since 2000	2000–2010/11
COWI Goss Gilroy Inc and EPOS Health Consultants 2007 [17]	Correlation	MDG 5.1; MDG 5.2	Supporting Sector-Wide Approach in the Health Sector	Tanzania	Multiple donors	Mixture of aid modalities, funding a sector-wide approach, with strong engagement with the Paris Principles.	Over USD 315 m in 2005	1999-on going
Edwards 2006 [18]	Causal	MDG 5.1	The Canada-China Yunnan Maternal and Child Health Project	China	Canadian International Development Agency	Project approach but the intervention involves co-planning and co-management with the local partner (alignment and ownership), so weak adherence to Paris Principles.	Unspecified	1997–2003
Magnani 1998 [19]	Causal	MDG 5.3	National Family Assistance Programme	Honduras	World Bank; USAID; other donors	Government-initiated programme with discrete donor project assistance (government ownership).	Unspecified	From 1990 (end date unspecified)
Mansour 2010 [20]	Correlation	MDG 5.1; MDG 5.5	Leadership Development Programme	Egypt	USAID (plus implementing partners)	Pilot project, funded by USAID, but requested by the country and then scaled up with strong local ownership (ownership).	Unspecified	2002–2003
Perks 2006 [21]	Correlation	MDG 5.1; MDG 5.2; MDG 5.3; MDG 5.5	The Sayaboury Programme	Lao People’s Democratic Republic	Save the Children Australia (with AusAID support)	Long-term technical assistance from an NGO, embedded within national administration. Indication of strong ownership and alignment.	USD 4 m over 12 years	1991–2003 (programme implemented in four 3-year stages)
Shiffman 2004 [22]	Correlation	MDG 5.1; MDG 5.2; MDG 5.5	Safe Motherhood	Honduras	Various donors (including USAID, IADB, UNFPA, World Bank, and other bilateral donors)	Intervention includes various aid modalities, with evidence of close partnership between government and donors leading to Safe Motherhood becoming a political priority (ownership).	USAID provided grants of USD 54 m between 1981 and 1988; then a further USD 57.3 m between1988 and 2000. IADB approved a USD 27 m loan in 1987. Other amounts unspecified.	1970s, 80s, 90s
World Bank 2003 [23]	Correlation	MDG 5.1; MDG 5.2; MDG 5.5	Comprehensive Maternal and Child Health Project (Health IV)		World Bank	Soft loan, requiring co-financing and government involvement, indicating a degree of local ownership of the intervention.	USD 90 m credit for total project cost of USD 139 m	1995–2002
World Bank 2006 [24]	Correlation	MDG 5.1; MDG 5.2; MDG 5.3; MDG 5.5; MDG 5.6	Fourth Population and Health Project (FPHP); Health and Population Project (HPPP)(renamed Health and Population Sector Program)	Bangladesh	World Bank (with 8 additional donors as co-financers)	Project but involving a pooled, basket fund with government co-funding.	WB provided USD 180 m for FPHP. Amount for HPPP unspecified	1992–1998; 1998–2005
World Bank 2007 [25]	Correlation	MDG 5.2; MDG 5.3; MDG 5.5	Ghana Second Health and Population Project (HPP II); Ghana Health Sector Support Project (HSSP); Second Health Sector Program Support Project (HSPSP)	Ghana	World Bank	Programme aid (credit/loan), turning into a Sector-wide approach, so good adherence to Paris Principles.	HPP II USD 18.9 m disbursed; HSSP USD 25.1 m; HSPSP USD 57.6 m loan and USD 32.4 m grant	1991–2007

No studies report just on general budget support.
Two studies [17], [22] cover a mixture of aid modalities, including budget support, sector budget support, pooled funding and projects.
Four studies pertain more specifically to a sector-based approach, including the use of sector budget support [17], [25], pooled funding [24], and multi-donor trust funds and silent partnerships [16].
Two studies concern project aid with clear alignment and ownership [18], [23]. The World Bank study on China [23] reports aid in the form of a soft loan with government co-financing. There is evidence of local ownership of the intervention, and this would appear to be a discrete project with on-budget aid which is on-plan. It is much harder to judge the extent to which the interventions adhere to the Paris indicators of ownership, alignment, harmonisation, managing for results and mutual accountability. However, within our included studies:
COWI et al. [17] engages most with the Paris Principles. The study assesses the effectiveness of harmonised support for the health sector in Tanzania under a sector-wide approach. The sector-wide approach (SWAp) involves more than 20 development partners and captures aid provided through different modalities (general budget support, health basket fund, health block grants, bilateral projects and programmes, funding from Global Health Initiatives). There is an indication of government ownership, alignment with national systems and strategies, and harmonisation of donor support.
Barnett et al. [16] provides another example of a harmonised approach, with the donor - DFID - working closely with and through other donors in support of the health sector in Indonesia. There is also close alignment with the government’s strategy.
Two World Bank studies [24], [25] describe the establishment of sector-wide approaches. The study on Bangladesh [24] implies there is strong country ownership and a harmonised approach by donors, with the World Bank and eight additional donors pooling funds into a special account which the Ministry of Health could access. The study on Ghana [25] outlines the shift over time from a project approach to pooled donor funding in support of a sector-wide approach in the health sector. There is limited information on aid management, but this programme conforms to many of the Paris Principles, including a harmonised approach and alignment with a nationally-owned strategy.
Four studies [19]–[22] demonstrate strong country ownership in terms of the initiation, management or sustainability of the intervention, although the information on the funding modalities is limited. Mansour et al. [20] provides an example where a discrete donor-funded project, requested by the Egyptian authorities but which was not on-budget, led to a nationally-owned, scaled up and successful programme.
The World Bank [23]–[25] interventions could be considered a sub-group as the funding is based on credits (soft loans) rather than grants, with projects requiring co-financing from local authorities and therefore by their nature have government involvement and hence a degree of alignment and ownership.

Of the Pool A studies, two presented data based on causal methodological designs and eight based on correlation designs.

Twenty of the studies concerned general aid (see table 2) [3], [26]–[45]. They fall into the following aid sub-categories:

**Table 2:** Characteristics of Pool B Studies: General Aid.

Study	Study Design	Outcomes Measured	Programme/Project Name	Country	Aid Donor/s	Analysis of aid modality and management	Funding Amount	Year of Intervention
Agha 2002 [26]	Causal	MDG 5.3	Social Marketing Adolescent Health Project	Cameroon; Botswana; South Africa; Guinea	USAID	Study describes discrete project inputs with no indication of adherence to Paris Principles	Unspecified	1994–1998
Baird 2010 [27]	Causal	MDG 5.2; MDG 5.4	Safe Motherhood Project	Indonesia	World Bank	The intervention is described as a first phase in a large-scale programme, suggesting partnership with the Indonesian Ministry of Health. However, there is insufficient detail to adequately assess depth of ownership or alignment, and the study describes discrete project interventions.	USD42.5 m	1998–2003
Barbey 2001 [28]	Causal	MDG 5.1; MDG 5.2; MDG 5.5	Dinajpur SafeMother Initiative	Bangladesh	CARE (Cooperative for Assistance and Relief Anywhere)	The intervention takes a project approach but it is implemented in conjunction with the Government of Bangladesh and UNICEF; hence a suggestion of harmonisation and a degree of alignment. However, there is no indication of funding being on-budget.	Unspecified	1999–2001
Buckley 2006 [29]	Correlation	MDG 5.3	Various	Uzbekistan	Various bilateral (incl. USAID) and multilateral agencies (incl. UNFPA, UNESCO, UNAIDS) and Non- Governmental Organisations	Study concerns multiple projects and donors; design is not conducive to determining any adherence to the Paris Principles	Unspecified	1993–2000
Campbell 2005 [30]	Correlation	MDG 5.1; MDG 5.2; MDG 5.3; MDG 5.5	Safe Motherhood Programme	Egypt	USAID	Project approach with a suggestion of alignment with a national strategy as the project provides support to the national Safe Motherhood programme. Insufficient to classify as Paris-style aid.	Unspecified	Series of projects running from 1985–2005
Debay 2007 [31]	Correlation	MDG 5.3	The Toliara Province Child Survival Project	Madagascar	USAID	Project approach using implementing partners; suggestion of government ministries as partners, but no indication of adherence to Paris Principles.	USD1,229,843	2003–2006
Hounton 2008 [32], [33]	Causal	MDG 5.1; MDG 5.2; MDG 5.5	The Skilled Care Initiative	Burkina Faso	Bill and Melinda Gates Foundation; Family Care International (implementing partner)	Project approach; insufficient information to indicate any adherence to the Paris Principles.	Unspecified	2001–2005
Mathur 2004 [34]	Causal	MDG 5.3; MDG 5.5	The Skilled Care Initiative	Burkina Faso	Bill and Melinda Gates Foundation; Family Care International (implementing partner)	Discrete project interventions, involving community participation, but no indication of adherence to the Paris Principles.	Unspecified	2001–2005
Meuwissen 2006 [35]	Causal	MDG 5.3	Unspecified	Nicaragua	UK Department for International Development (DFID); Central American Health Institute (implementing partner), plus 4 NGOs (implementing partners)	Project with insufficient information to assess adherence to Paris principles.	Unspecified	Dates unspecified but vouchers distributed between Sept 2000 and July 2001
Mize 2008 [36]	Correlation	MDG 5.2; MDG 5.5	Child Survival Grant	Timor Leste	USAID	Project approach using implementing partners, with Ministry of Health as a partner (suggestion of alignment); insufficient detail to assess adherence to the Paris Principles.	Unspecified	2004–2008 (on-going to 2010)
Mulay 1992 [37]	Correlation	MDG 5.3	3 projects: National Integrated Medical Association; Centre for Matru Mandir; Yusuf Meherally Centre	India	Various state bodies, national and international NGOs	NGO-led projects, with no information about aid flows and management.	Unspecified	Since 1986
Options Consultancy Services Ltd 2010 [38]	Correlation	MDG 5.1; MDG 5.2; MDG 5.5	Support to Safe Motherhood Program	Nepal	DFID (main donor); five implementing partners (John Hopkins University, Actionaid, Ipas, UN Mission to Nepal, UNICEF)	Suggestion of alignment (with national strategy) as the project provides support to the national Safe Motherhood programme with some co-funding from government; but insufficient information on the funding mechanisms and management to classify intervention as ‘Paris-style’, i.e. unclear if funding is budget support or sector budget support, if it is on-budget or using national systems.	Limited information on overall budget, but Rs. 820 m in financial aid 2009/10 for Options Technical Assistance	2004–2010
Powell-Jackson 2006 [3]	Correlation	MDG 5.1	Not relevant (article provide an overview of donor giving patterns)	Covers approximately 150 countries	OECD-DAC donors	Data on different aid modalities (including budget support) but the study does not seek to make claims on behalf of different types of aid, nor does it provide detail to enable a classification as Paris-style aid.	Amounts reported in Creditor Reporting System for majority of donors	Not relevant
Price 2009 [39]	Correlation	MDG 5.2; MDG 5.3; MDG 5.5	Unspecified	Rwanda	PEPFAR (plus implementing partner Family Healthcare International)	Suggestion of ownership as direct grants are provided to local Primary Health Centres; but insufficient information to assess adherence to Paris Principles.	USD 63,000 for basic HIV care from FHI in year one going down to USD 32,000 a year after that	Unspecified
Ronsmans 2001 [40]	Correlation	MDG 5.2	Safe Motherhood Programme	Indonesia	MotherCare (USAID-funded initiative)	Suggestion of alignment (with national strategy) as the project provides support to the national Safe Motherhood programme in partnership with the Ministry of Health; but insufficient information on the funding mechanisms and management to classify the intervention as ‘Paris-style’.	Unspecified	1994-(end date unspecified)
Senlet 2008 [41]	Correlation	MDG 5.2; MDG 5.5	PAIMAN Project	Pakistan	USAID (plus implementing partners)	Discrete project with suggestion of some government involvement.	USD 49,943,857 through 2009	2005–2009
Snyder 2003 [42]	Correlation	MDG 5.3	Not relevant (study presents meta-analysis).	Covers multiple countries	USAID (funder); John Hopkins University (implementing partner)	Study analyses multiple targeted projects; limited information about the aid flows and management to enable classification as Paris-style aid.	Unspecified	Not Relevant
Williams 2007 [43]	Causal	MDG 5.3	Africa Youth Alliance Programme	Ghana, Tanzania, Uganda	Bill and Melinda Gates Foundation (funder); United Nations Population Fund (implementing partner); Pathfinder International (implementing partner); Program for Appropriate Technology in Health (implementing partner)	Project approach with suggestion of partnership with government, but limited information to assess adherence to the Paris Principles.	Unspecified	2000–2006
World Bank 1998 [44]	Correlation	MDG 5.2	Family Health Projects I & II, Sexually Transmitted Infections Project	Zimbabwe	World Bank	Insufficient information to suggest anything other than discrete projects.	Unspecified	Initial loan provided in 1986, second one in 1991, then separate project on Sexually Transmitted Infections launched in 1993
World Bank 2008 [45]	Correlation	MDG 5.3; MDG 5.6	Egypt Population Project	Egypt	World Bank	Programme aid (credit/loan), but most of the information is about support for NGOs with insufficient information to assess adherence to the Paris Principles.	USD17.2 m	1998–2005

Six studies [29], [32], [33], [35], [38], [44], [45] relate to project or programme aid. However because the information on aid is very limited or unclear, we have insufficient means to ascertain whether the intervention(s) could be determined to be Paris-style.
Nine studies [27], [28], [30], [31], [36], [39]–[41], [43] relate to discrete projects which demonstrate some very limited adherence to the Paris Principles, e.g. they mention partnerships with national authorities indicating a degree of alignment with national strategies or a degree of ownership. Again however, there is insufficient information to suggest a more robust adherence to the Paris Principles.
Three studies [26], [34], [37] provide no evidence of adherence to any of the Paris Principles.
One study [27] mentions the aid intervention in relation to a broader development programme; however, the study itself focuses on discrete projects.
The objectives of three studies [3], [29], [42] are not conducive to analysing adherence to the Paris Principles, i.e. they are comparative studies relating to a large range of donors or projects.

Of the Pool B studies, nine studies presented data based on a causal design and eleven based on a correlation design.

The MDG 5 indicators addressed in the Pool A and Pool B studies were as follows.

- Maternal mortality ratio or rate (MDG 5.1): n = 12 [3], [17], [18], [20]–[24], [28], [30], [32], [33], [38].
- Births attended by skilled birth personnel (MDG 5.2): n = 17 [16], [17], [21]–[25], [27], [28], [30], [32], [33], [36], [38]–[41], [44].
- Contraceptive prevalence (MDG 5.3): n = 15 [19], [21], [24]–[26], [29]–[31], [34], [35], [37], [39], [42], [43], [45].
- Adolescent birth rate (MDG 5.4): n = 1 [27].
- Ante-natal care coverage (MDG 5.5): n = 14 [20]–[25], [28], [30], [32]–[34], [36], [38], [39], [41].
- Unmet need for family planning (MDG 5.6): n = 2 [24], [45].

The majority of the studies did not engage directly with MDG 5 terminology.

The studies covered 27 countries and aid interventions from bilateral donors, multilateral donors and non-governmental organisations. Most of the studies included in the review were evaluation reports and progress reports conducted by donors or commissioned by donor agencies, and not peer-reviewed publications. Bias arising from authorship is likely. Many of these were based on case studies of individual projects or countries from a small number of developing countries and aid donors. No studies were included which looked only at general budget support or sector budget support. The studies which captured most clearly the Paris Principles were the four which evaluated sector-wide approaches in health, which covered a range of aid modalities including sector budget support and, indirectly, general budget support [16], [17], [24], [25].

### Overview of Quality Assessment (Tables 3 and 4)

**Table 3:** Pool A studies quality assessment ratings.

	Independence of study	Reporting on aid intervention	Reporting on study design and methods	Robustness of the data analysis	Reporting on confounding factors
Barnett 2007 [16]	Medium	Medium	High	Low	Low
COWI Goss Gilroy Inc and EPOS Health Consultants 2007 [17]	Medium	High	High	High	Low
Edwards 2006 [18]	High	Medium	Low	Low	Low
Magnani 1998 [19]	Medium	Medium	High	High	Low
Mansour 2010 [20]	Low	Medium	Low	High	Low
Perks 2006 [21]	Low	High	High	High	High
Shiffman 2004 [22]	High	Low	High	High	High
World Bank 2003 [23]	Low	High	Medium	High	Medium
World Bank 2006 [24]	Low	High	Low	High	High
World Bank 2007 [25]	Low	High	Medium	High	Medium
TOTALS (n = )					
High	2	5	5	8	5
Low	5	1	3	2	3
Medium	3	4	2	0	2
Not answered	0	0	0	0	0

**Table 4:** Pool B studies quality assessment ratings.

	Independence of study	Reporting on aid intervention	Reporting on study design and methods	Robustness of the data analysis	Reporting on confounding factors
Agha 2002 [26]	Medium	Low	High	High	High
Baird 2010 [27]	High	Low	High	High	High
Barbey 2001 [28]	Medium	Medium	High	High	Low
Buckley 2006 [29]	High	Low	Medium	Medium	Medium
Campbell 2005 [30]	Medium	Low	High	High	Medium
Debay 2007 [31]	Medium	Medium	High	High	Low
Hounton 2008 [32], [33]	Medium	Low	Medium	Low	Low
Mathur 2004 [34]	Medium	Medium	High	Low	Low
Meuwissen 2006 [35]	Medium	Low	High	High	Medium
Mize 2008 [36]	Low	Medium	High	Medium	High
Mulay 1992 [37]	High	Low	Medium	High	Low
Options Consultancy Services Ltd 2010 [38]	Low	Medium	Low	Medium	Low
Powell-Jackson 2006 [3]	Medium	Medium	High	High	Low
Price 2009 [39]	Low	Low	High	Medium	High
Ronsmans 2001 [40]	Medium	Low	High	High	High
Senlet 2008 [41]	Low	Medium	High	High	Low
Snyder 2003 [42] *	Medium	Low	___	___	___
Williams 2007 [43]	Low	Medium	High	High	Medium
World Bank 1998 [44]	Low	Low	High	Low	High
World Bank 2008 [45]	Low	Medium	Medium	High	Medium
TOTALS (n = )					
High	3	0	14	12	6
Low	7	11	1	3	8
Medium	10	9	4	4	5
Not answered	0	0	1	1	1

* Snyder et al. reports a ‘meta-analysis’ from several data sets; there is no systematic review preceding the meta analysis.

The majority of studies were deemed to be of medium (n = 13 [3], [16], [17], [19], [26], [28], [30]–[35], [40], [42]) or low quality (n = 12 [20], [21], [23]–[25], [36], [38], [39], [41], [43]–[45]) for their study independence, suggesting that they were in some way related to the aid donor or the aid-funded project that served as the focus of the study. Most of the studies were rated as medium (n = 13 [3], [16], [18]–[20], [28], [31], [34], [36], [38], [41], [43], [45]) or low quality (n = 12 [22], [26], [27], [29], [30], [32], [33], [35], [37], [39], [40], [42], [44]) on the basis of their reporting of the aid intervention; moreover, their focus tended to be on the activities funded by aid and not the amounts of aid, the mechanisms through which it was donated, or the management of the funding. The majority of studies were rated as high (n = 19 [3], [16], [17], [19], [21], [22], [26]–[28], [30], [31], [34]–[36], [39]–[41], [43], [44]) or medium quality (n = 6 [23], [25], [29], [32], [33], [37], [45]) on the basis of their reporting on study design and methods. The majority of studies (n = 20 [3], [17], [19]–[28], [30], [31], [35], [37], [40], [41], [43], [45]) were rated as high quality on the basis of the robustness of data analysis. Only nine studies were rated as high quality for their reporting of confounding factors [21], [22], [24], [26], [27], [36], [39], [40], [44]; given the wide range of factors which can affect maternal and reproductive health outcomes this is a concern.

### Data Synthesis

It was not possible to aggregate or pool data from the studies in this review because the studies were different in design and the data they present. We therefore configured a narrative synthesis based on the data from each study. In the full review report we present tabulated results of the characteristics of the included studies, then the outcome data for each Pool [9]. Our synthesis brings together the findings from the synthesis of outcome data, the analysis of aid interventions in relation to outcomes, and the quality assessment findings to present: findings on the impact of Paris-style aid on MDG 5 outcomes, and findings on the impact of general aid on MDG 5 outcomes.

### Pool A Synthesis Results: Evidence of the Impact of Aid Delivered under the Paris Principles on MDG 5 Outcomes

#### MDG 5.1

One causal study providing a graphic presentation of data showed a decline in MMR over a five-year period (1997–2002) in China [18]. Data for MMR from five correlation studies found evidence that a reduction in MMR had occurred over time:

In a study conducted in Egypt, a locally owned leadership development programme in Aswan Governorate contributed to a reduction in MMR from 85/100,000 in 2005 to 35.5/100,000 in 2007. This signified a much greater reduction than that obtained in similar governorates in Egypt [20].
The Sayaboury Programme trained birth attendants in 495 villages in Lao People’s Democratic Republic. Over a five-year period the MMR was reported to have almost halved [21].
Reported outcomes from the Safe Motherhood Programme in Honduras found MMR reduced from 182/100,000 in 1990 to 108/100,000 in 1997 [22].
The Comprehensive Maternal and Child Health Project (Health IV) in China provided training for village birth attendants and improvements in basic mother and child health care. A reduction in MMR from 203.8/100,000 in 1992/3 to 69.6/100,000 per live births in 2001 was reported [23].
The Fourth Population and Health Project (FPHP) in Bangladesh provided support to family planning activities and produced divergent MMR data over three time periods: between the early and late 1990s the MMR showed an increase from 485/100,00 to 499/100,000 per live births but this reduced to 400/100,000 per live births by the early 2000s [24].

A single correlation study which reported an increase in MMR was conducted in Tanzania where a multi-donor funded sector-wide approach in the Health Sector demonstrated an increase from 529/100,000 in 1996 to 578/100,000 in 2004/5 [17].

Reporting on study design/methods, data analysis and confounding factors was generally good. Three studies scored high on these quality assessment criteria [17], [21], [22]. Only one study scored low on all three [18].

#### MDG 5.2

No Pool A causal studies reported on this indicator. The review found seven correlation studies which reported aid delivering a higher proportion of births attended by skilled health personnel:

In Indonesia, a programme funded by the UK’s Department for International Development reported a 30.5% increase in the percentage of attended births (from 41% in 2000 to 71.5% in 2004). Few details of the nature of the intervention were reported [16].
An aid-funded programme in Tanzania reported an increase in attended births from 36% in 1999 to 46% in 2004/5 [17].
One study showed an increase in the proportion of attended births in the northern districts of Lao’s People’s Democratic Republic between 1999 and 2003 [21].
One study documented an increase in the percentage of institutional deliveries from 45% in 1989/90 to 61% in 1998 [22].
A report by the World Bank [23] published data showing a hospital delivery rate in China which increased from 18.6 in 1992–3 to 59.6 in 2001.
Data collected at three time points after the implementation of a strategy to train Female HAS Assistants in the fourth Population and Health Project (FPHP) in Bangladesh demonstrated modest increases in the percentage of attended births from 5.2% in 1996/97, 7.1% in 1999/2000 and 7.5% in 2004 [24].
The Ghana Second Health and Population Project (HPP 11) produced longitudinal data for percentages of attended births between 1988 (40.2%) and 2006 (49.7%); however it should be noted that the definition of what constituted a ‘skilled worker’ changed over that time frame [25].

Reporting on the study methods, data analysis and confounding factors was generally good, with one exception [18]. Four of the studies scored low on study independence [21], [23]–[25].

#### MDG 5.3

In an evaluation of cash coupons in Honduras, Magnani et al. [19] – the one causal study reporting on this indicator in Pool A - reported changes in contraceptive use as coefficients from a logistic regression analysis but no statistically significant differences were observed between those who received the coupons and those who did not.

Three correlation studies reported higher rates of contraceptive use and family planning following the aid-funded intervention:

The Sayaboury Programme in Lao People’s Democratic Republic reported an increase in contraceptive use from 12% in 1998 to 67% in 2003 [21].
A 2006 report by the World Bank observed that the percentage of women using family planning services in Bangladesh increased between 1993/4 (44.9%) and 2004 (58.5%) [24].
A report by the World Bank in 2007 presented data from Ghana which showed an increase in the use of modern methods of contraception used by married women from 5.2% in 1988 to 11.5% in 2006. The proportions of married women using any method of contraception over the same time period were even greater, increasing from 5.2% in 1988 to 13.6% in 2006 [25].

Reporting on study design/methods, data analysis and confounding factors was generally good. The three correlation studies scored high on their reporting on the aid intervention.

#### MDG 5.4

We found no studies that reported on Paris-style aid in relation to the adolescent birth rate.

#### MDG 5.5

We found no causal studies in Pool A which reported on this indicator. Interventions delivered with Paris style aid were associated with increases in ante-natal care (ANC) coverage in six correlation studies:

In the year after an aid-funded intervention ended in Egypt, the number of prenatal visits per woman was reported to have increased from 1.3 to 3.7 in Aswan governorate [20].
An aid-funded programme in Lao People’s Democratic Republic was thought to have contributed to the proportion of pregnant women attending three ante-natal clinic visits, which increased from 24% in 1997 (in six districts) to 58% in 2003 (in 10 districts), compared with 20% nationwide [21].
During the Safe Motherhood Programme in Honduras there was an increase in the number of women aged 15–44 years who had at least one ante-natal care visit with medically trained personnel, from 72% in 1989/90 to 85% in 1998 [22].
A report by the World Bank published baseline estimates collected in China (1992/3 = 22, 1995 = 47.4) and a follow-up estimate of 84.2 in 2001. It is unclear as to what these data pertain (percentages, means or absolute numbers) [23].
A second World Bank report published data to show increases in the percentages of ante-natal visits after targeted interventions in Bangladesh. Between 1996/97 and 2004 the percentage increased from 19.6% to 31.2% [24].
A third report by the World Bank published data to show that over the course of two World Bank projects implemented back-to-back in Ghana, the number of ANC visits remained consistently high. While the time series data would suggest an increase in overall coverage (from 82.4% in 1988, to 87.5% in 1998 and 91.9% in 2003), the fact that the exact wording of the question used to solicit this data was changed over the time period means direct comparisons are not possible [25].

Reporting on the study design/methods, data analysis and confounding factors was generally good, with one exception [20]. Five studies scored low for study independence [20], [21], [23]–[25].

#### MDG 5.6

No Pool A causal studies reported on this indicator. Our review identified one correlation study which reported that the percentage of women with an unmet need for family planning in Bangladesh decreased from 18.1% in 1993/4 to 11.2% in 2004 [24]. This study scored low on study independence and reporting on the research design/methods but high on all other quality assessment criteria [24].

### Pool B Synthesis Results: Evidence of the Impact of General Aid on MDG 5 Outcomes

#### MDG 5.1

The MMR data in two causal studies were varied; both used proxy indicators for MMR:

The first, which examined the effects of the Skilled Care Initiative in Burkina Faso, used the risk of pregnancy-related mortality as a proxy for MMR. It observed that the project’s efforts to increase the rates of skilled attendance at births in project districts did not demonstrate a statistically significant difference in the risk of pregnancy-related mortality in women aged 15–49 who participated in the project, compared with those who did not [32], [33]. The authors did observe, however, that attending ante-natal care was associated with a statistically significant reduction in the risk of pregnancy-related mortality.
A second causal study used Emergency Obstetric Care (EmOC) as a proxy for MMR (assuming that EmOC, if received in an upgraded facility, would not result in maternal death). Accordingly, it found a higher percentage of women with complications used EmOC in the intervention arm of the study than in the control groups (16% to 39.8% versus 12.5% to 25.5% and 11.1% to 12.1%) over a seven year period [28].

Data from three correlation studies were likewise varied:

One study using a correlation design reported reductions in MMR between 1992/3 and 2000 and evaluated outcomes from three USAID-funded projects which are thought to have supported the Safe Motherhood Project in Egypt between 1985 and 2005. Between 1992–3 and 2000 the MMR dropped from 174/100,000 to 84/100,000 [30].
The data from a second correlation study on MMR was unclear [38].
Data from a third correlation study was depicted graphically and was intended to demonstrate a positive association between MMR and official development assistance per head [3].

Reporting on study design/methods, data analysis and confounding factors across the studies was mixed, with no single study scoring high or low on all three criteria. Only one study scored low on study independence [38].

#### MDG 5.2

Reports on attended births from three causal and six correlation studies contain data which consistently reported higher proportions of attended births.

Causal studies:

An evaluation of the Safe Motherhood Project in Indonesia found statistically significant increases in the percentage of attended deliveries between baseline and follow-up periods within intervention and control groups, but no statistical difference between the groups was observed [27].
In Bangladesh, the percentage of total births taking place in facilities increased over the life of the Dinajpur Safe Motherhood Initiative: in the intervention area from 2.4% to 10.5%; in the upgraded comparison area A: from 7.2% to 12.1%; and in the control area: from 4.5% to 5% [28].
Hounton et al. [32], [33] reported the pregnancy-related mortality risk decreased with increasing proportions of women attending ante-natal care (P = 0.032) or giving birth in an institution (P = 0.065) in Burkina Faso.

Correlation studies:

The Safe Motherhood Programme in Egypt contributed to a 50% increase in the percentage of deliveries presided over by a skilled attendant from 40.7% in 1992/3 to 60.9% in 2000 [30].
Mize et al. [36] reported an increase in the percentage of children (aged 0–23 months) whose last delivery was assisted by a skilled birth attendant in programme districts. However, district level data for 2006, 2007 and 2008 show the mean level of monthly home deliveries in the Remexio district increased, while the same data for the Maubara district showed a decline in attended deliveries.
An evaluation of the Safe Motherhood Project in Nepal reported that facility deliveries increased by 2% per year but the exact time period to which this refers is unclear [38].
Price et al. [39] reported an increase in the number of births at a health care facility in Rwanda, in a project designed to improve health care for people with HIV (from 5 to 219). The time periods for these data are unclear.
One report showed an increase in the number of attended births from 37% to 59% over the period 1993/6–1999 in Indonesia [40].
An early evaluation of the PAIMAN project in Pakistan detected a small increase in the number of attended births between 2005 (35%) and 2007 (38%) [41].

The data from a seventh correlation study were presented graphically with no absolute numbers available. The graph suggests little change between 1986 and 1993 in Zimbabwe for maternity admissions [44].

Reporting on study design/methods, data analysis and confounding factors was generally good, with a few exceptions [32], [33], [38], [41]. The studies scored less well on reporting on the aid intervention and study independence, with four studies scoring low on independence [36], [38], [39] and five scoring low on aid reporting [27], [30], [32], [33], [39], [40].

#### MDG 5.3

Four causal studies demonstrated mixed results from aid-funded interventions aimed at increasing contraceptive use:

A USAID-funded social marketing project targeting adolescents in four countries used peer education, peer educators, youth clubs, and mass media advertising to promote safe sexual health practices. Data from Cameroon, Botswana, South Africa and Guinea revealed that only in Cameroon did the numbers of women who had ever used a condom differ to a statistically significant level from the comparator group (2.27 as opposed to 0.87). Similarly the numbers of people who used a modern method of contraception for pregnancy prevention only differed to a statistically significant level in Cameroon [26].
A study based in Nepal evaluated the effect of three interventions designed to improve reproductive health. No statistically significant differences were observed between baseline and the end of the study [34].
A study investigating the effect of vouchers for reproductive health clinics in Nicaragua found the use of family planning methods were statistically significantly different between those who received the vouchers and those who did not (1.33, (95% CI 0.77 to 2.29)), as was the prevalence of condoms used in the last sexual contact (1.84 (95% CI 1.11 to 3.03)) [35].
One study found a statistically significant impact on condom usage amongst females involved in the African Youth Alliance Programme in Ghana, Tanzania and Uganda, when compared with females outside the intervention, on four condom-related indicators (condom at first sex, condom at last sex, ever used a condom with current partner, and always use condom with current partner). A less favourable impact was demonstrated amongst study males, and just two of the four impact indicators were deemed statistically significant for the project males of Tanzania [43].

Seven correlation studies reported positive changes in contraceptive prevalence:

The prevalence of the use of modern methods in Uzbekistan was shown to increase from 28% in 1989 to 60% in 2002. No data from significance tests were presented in the report and it is unclear if these data are statistically significantly different between time periods [29].
In an evaluation of the Safe Motherhood Programme in Egypt the contraceptive prevalence was shown to increase between 1992/3 (47.1%) and 2000 (56.1%). No significance tests results were presented in the report and it is unclear if these data are statistically significantly different between time periods [30].
In an evaluation of the Toliara Province Child Survival Project in Madagascar the percentage of mothers who were not pregnant and did not want another child in the next two years and were using a modern method of contraception was observed to increase across the project sites from 9% to 24% (95% CI 19%–29%). The outcome data specific to Betioky district demonstrated an increase from 17% to 28% (95% CI 23–33%). No baseline data were presented for Toliara II district but the final follow-up estimate is similar to that observed in Betioky, odds ratio; 24 (95% CI 19 to 29) [31].
An assessment of the family planning performance of three non-governmental organisations in India found the couple protection rate increased between 1986 and 1991, the range was from 6.9% to 58.7% [37].
The number of new family planning acceptors increased over two time periods in a study based in Rwanda; total baseline estimate was 100 (mean) which increased to 155 (mean). An even greater increase was observed in the uptake of family planning services amongst those at highly active antiretroviral therapy sites 24–126 mean [39].
In a meta-analysis of USAID data from electronic data sets, Snyder et al. [42] found the pre-intervention use of contraception was lower than the post-intervention use of modern family planning methods. When data for traditional methods were included in the analysis the increase was more marked. The nature of the interventions and the exact locations from which the data were collected are not well described.
In a report by the World Bank [45] on the Egypt Population Project an increase in the use of contraceptives from 40.2% to 45.2% was observed between 2000 and 2005.

Reporting on study design/methods, data analysis and confounding factors was generally good, with causal studies scoring higher on these criteria. Six studies scored low on reporting of the aid intervention [26], [29], [30], [37], [39], [42].

#### MDG 5.4

We found one causal study which reported indirectly on the adolescent birth rate. In the Safe Motherhood Project in Indonesia, the percentage of teenage pregnancies increased between the baseline and follow-up period in both the intervention and control groups but in neither case was the increase shown to be statistically significant [27]. This study scored high on all but one of our quality assessment criteria, reporting on the aid intervention, for which it scored low.

### MDG 5.5

Three causal studies reported on changes in ante-natal care (ANC) coverage:

In an evaluation of the CARE supported Dinajpur SafeMother Initiative the percentage of women receiving ante-natal care in the intervention group in Birampur demonstrated the largest increase between baseline and follow-up periods (from 2.4% to 10.5%) whilst the comparison area of Debiganji demonstrated the smallest increase (from 4.5% to 5%). It is unclear if these differences have arisen by chance [28].
Hounton et al. [32], [33] found that increasing ante-natal care coverage was shown to be associated with a statistically significant reduction in the risk of pregnancy-related mortality (P = 0.032).
The access of rural females to formal ante-natal care during their first pregnancy was not influenced by the Engender Health project in Nepal [34].

Five correlation studies implied an increase in ANC coverage as a result of aid-funded interventions:

The number of women who received any ante-natal care in Egypt failed to increase between 1992/3 and 2000 in Egypt (remaining constant at 52.9%), although the Campbell et al. [30] study, which cites these figures, concurrently reports a 35% increase in ANC coverage over the period, calling into question the veracity of data cited.
Aid funded improvements in health care infrastructure in Rwanda may have been responsible for the observed increase in the total number of new ante-natal clients, the coverage rate of new ante-natal care clients and all four ante-natal visits completed between the baseline and follow-up periods in a study by Price et al. [39]. It is unclear, however, if these estimates arose by chance as no significance tests were reported.
The PAIMAN project in Pakistan reported modest changes in the percentages of women who received three or more ante-natal visits during their last pregnancy between 2005 (27%) and 2008 (35%) [41].
One study pointed to an increase in the percentage of mothers of children age 20–23 months who had received one or more ante-natal care visits during their last pregnancy in programme districts in Timor Leste; the findings are based on estimate figures however [36].
One study showed an apparent increase in the up-take of ANC visits (from 45% to 60% at end-line) for an ActionAid initiative in Nepal [38].

The quality of the studies was mixed: reporting on the study design/methods and data analysis was generally good but five of the eight studies scored low in the reporting of confounding factors [28], [32]–[34], [38], [41]. Four of the eight scored low on study independence [36], [38], [39], [41].

#### MDG 5.6

We found one correlation study which reported an increase in met need for family planning after the aid intervention. Here, the met demand for family planning increased slightly in rural Upper Egypt from 69% in 2000 to 73% in 2005, but was virtually unchanged for Egypt as a whole (84% in 2000 and 85% in 2005). Only Upper Egypt and the Frontier Governorates documented improvements in meeting existing demand, rising from 74% to 78% and 75% to 85%, respectively. The demand of women with no education was the least satisfied (at 81%), while those with some, primary education (87%) and (86%) or (88%) secondary education was 87%, 86% and 88% respectively [45]. This study scored high on study independence and data analysis, and medium on all other criteria [45].

### Overview of Synthesis Findings

Viewed together the 30 included studies suggest that aid interventions, whether delivered using the Paris Principles or not, might be associated with some positive changes in the target areas of intervention as demonstrated by changes in the outcome data. However, this conclusion should be interpreted with caution as the claims are of association rather than causality. Data are not comparable across the studies, which cover different countries and time periods, and reporting on confounding factors and alternative explanations is generally weak. Therefore we cannot be confident that changes are happening because of the manner in which aid is delivered. The data do not allow for a meaningful comparison between aid delivered according to the Paris Principles and aid delivered outside this framework.

## Discussion

### Summary of Evidence

This review presents the first attempt to review systematically the publicly available literature on the impact of general aid and aid delivered under the Paris principles on MDG 5 outcomes.

An initial yield of 211 reports was screened to produce a total of 30 studies for synthesis. Of these, ten of the studies concerned Paris style aid and 20 concerned general aid. Using the six MDG 5 indicators as outcome variables, the review finds that aid interventions may be associated with small improvements in maternal and reproductive health outcomes. However, the data do not allow for a comparison between the outcomes associated with Paris style and general aid.

The review identified discernible gaps in the evidence base on aid interventions aimed at addressing MDG 5, notably on indicators MDG 5.4 (adolescent birth rate) and 5.6 (unmet need for family planning).

### Limitations

The question demanded a focus on studies presenting statistical evidence of impact. We did not identify any experimental studies, and excluded all that were based on purely qualitative research design. Qualitative research methods are increasingly used in the design of impact evaluation studies in international development [46], and since this review was originally commissioned there has been considerable reflection on how to include a broader spectrum of study design methods within impact evaluation. This question may have yielded more results if a broader approach has been taken regarding study design at the outset.

Our review was conducted under considerable time pressure which prevented us from making contact with the authors of included studies. It is possible the analysis could be extended by eliciting more information about the studies.

We adopted a broad approach to defining the MDG 5 indicators, for example including studies which used proxy measures for some of the indicators. This was appropriate as the majority of studies failed to engage with the MDG 5 terminology, while in several studies the outcome indicators for which data were collected changed over the course of the reporting period [16], [25]. Even with a generous application of the MDG definitions, the synthesis reveals gaps in the evidence base on maternal and reproductive health.

Detail on aid modalities, flows and management was limited in many of the included studies. Our search identified only eight studies that reported on interventions which concluded or began after the adoption of the Paris Declaration in 2005 [16], [17], [25], [31], [36], [38], [41], [43]; and the data in many of the studies predated the adoption of the Paris Declaration. With the Paris Principles being so recent, many of the studies did not engage fully with the ideas of the Paris Declaration. It was appropriate therefore to take a broad approach to defining Paris-style aid as any intervention which showed clear adherence to all or some of the Paris Principles, including studies which predated the Declaration itself. This approach recognises that the Paris Declaration was not the beginning of a new approach, but rather a milestone in a longer policy process dating back to the mid-1990s to reform aid both in terms of outcomes (a poverty focus leading to the adoption of the MDGs in 2000) and in terms of effectiveness. Moreover, taking a longer time-span also facilitated the comparison dimension of the review. Still, the best we could deduce were degrees of adherence to one or more of the five Paris Principles. This highlights the constraints in analysing the impact of the Paris Declaration when research does not engage directly with it or give information on aid management. It was much harder to discern whether an intervention could be considered to be adhering to the Paris Principles than anticipated.

Studies using correlations designs were the most common picked up in our review. We are aware that randomized control trials have been conducted on maternal and reproductive health in developing countries [47]. Likewise, studies exist which appear to show global reductions in MMR [1]. However, such studies would not meet our inclusion criteria because the focus of this review is on the impact of particular types of aid intervention.

### Conclusions

The use of systematic reviews for helping development agencies make sense of their policies, decisions and investments is growing. Until relatively recently there was little political impetus to attempt to prove the impacts of different types of development aid upon development outcomes. This review was commissioned against a particular political back-drop in the UK: on the one hand the increased focus on value for money in public policy and on evidence-based policy; on the other hand the run-up to the 4^th^ High Level Forum on Aid Effectiveness in November 2011, at which point evidence of the impact of new aid modalities was under scrutiny within a longer process of attempts to make aid more transparent, more accountable and more efficient.

This review addressed a particularly complex development question, namely the impact of a hands-off approach to aid delivery upon health outcomes. The Paris Principles seek to place greater responsibility in the hands of local partners and focus on enhancing the environment in which aid is used rather than on achieving tangible objectives with aid inputs. It is perhaps not surprising that we found few studies which responded directly to this question. We observe that the aid effectiveness literature tends to focus on the aid and policy side, and much of the literature on health focuses on providing and analysing information on health alone. Studies which do both are rare.

The review consequently highlights gaps in the public reporting of the evidence for aid effectiveness in relation to health outcomes. In particular, the review identified discernible gaps in the evidence base on the impact of aid on MDG 5 which are of concern if these targets are to be met by 2015, notably on indicators MDG 5.4 (adolescent birth rate) and 5.6 (unmet need for family planning). We recognise that there is a considerable body of literature which assesses maternal and reproductive health outcomes; what this review demonstrates is that rigorous, independent studies which have been subject to peer-review and which attempt to make a plausible association between the mode of aid delivery and those health outcomes are extremely limited. Studies on health outcomes need to provide more complete details about the aid intervention as well as outcome data if a question such as this is to be answered.

It was informative that the studies in this review which conformed most closely to the Paris principles reported on sector-wide approaches in health. Reflecting back on our use of aid modalities as proxies for the Paris Principles, the sector-wide approach might make the best proxy for Paris-style aid when looking at outcomes such as maternal health. That said, it is also insightful that the included studies on sector-wide approaches were cautious about making claims on behalf of aid. This highlights the difficulty in disaggregating cause, effect and impact in the highly complex environment of non-project aid. It becomes contradictory to try and attribute results to individual aid inputs when working with multiple partners and through national systems. Such limitations are recognised in the literature evaluating aid effectiveness [48]. We would consequently recommend using aid modalities or management systems rather than the Paris Principles in evaluating aid interventions.

Furthermore, the review also highlights the time-lag between the adoption of a policy like the *Paris Declaration* and a) the production of evidence of effectiveness that is data driven, and b) the translation of the policy into demonstrable changes in outcomes, especially when aid is a small part of the overall picture of social transformation.

Regarding the maternal and reproductive health interventions described in our studies, it is clear that before claims about cause and effect in the field can be made we need robust baseline data to couple with later data. Many of the studies had an inadequate statistical base, often without good time series data, too short a timeframe to estimate changes when any impact is likely to have a longer lead time, and a lack of attention to context. Additionally, several of the studies were based on data around institutions, and changes at that level cannot be judged unless there are data on what is going on external to those institutions. There are other factors that affect maternal and reproductive health and we are concerned that the focus on healthcare inputs to tackle apparently ‘medical’ problems (with hence a focus on medical outcomes) inevitably marginalises the political, social and economic factors, including gender politics, which influence maternal health outcomes. Reconceptualising maternal and reproductive health as a complex socio-political field (with a biological component) would make interventions more responsive to socio-political context and less medically driven. The implications of the above are that research to evaluate the impact of aid interventions on maternal and child health need to engage with social and economic processes beyond the confines of health-care institutions. Women’s general health as well as health during pregnancy and the post-partum period is affected by processes in the wider national and global economy as well as by micro-political processes within the household. Massive changes in the economy–for instance, land fragmentation, shifts in the balance of agricultural production towards export crops and away from domestic consumption, retrenchment or expansion of off-farm employment opportunities for women as well as men, demographic processes that affect the size of the working-age population–all affect the capacity of households to sustain viable livelihoods and thereby maintain good health for all their members. At the same time, processes within households–such as gender-biased decision-making about entitlements to food and curative medical care, work burdens and the capacity of women to take time off to recuperate after childbirth or to consult medical practitioners–will affect women’s health status. In combination, such processes may compromise or reinforce the impact of aid that aims to improve maternal and child health outcomes primarily via expansion of institutional deliveries or expansion of family planning services. Without adequate baseline data on such wider processes and without adequate assessment of how such confounding factors themselves are changing, attempts to evaluate the impact of targeted aid are seriously flawed by their conceptual and methodological limitations.

The use of systematic reviews in the evaluation of international development is new, and we found the process challenging because aid interventions, such as budget support, generally are not designed with transparent outcomes in mind; their purpose is to strengthen a government’s ability to implement policy. Researching the impact of this type of intervention needs to focus primarily on whether an aid- strengthened government budget results in better development results than a non-aid-strengthened government budget.

However, systematic review methods may be valuable in informing new research and methodological approaches in international development, not least because they identify gaps in the evidence base. This may encourage donors towards more transparent ways of working regarding decision-making, policy and impact evaluation. This systematic review highlights the gaps in our understanding about the impact of international aid and has an important role to play in the design and conduct of future research.
